# Axial Compressive Behaviours of Coal Gangue Concrete-Filled Circular Steel Tubular Stub Columns after Chloride Salt Corrosion

**DOI:** 10.3390/ma17112782

**Published:** 2024-06-06

**Authors:** Tong Zhang, Hongshan Wang, Xuanhe Zheng, Shan Gao

**Affiliations:** 1 School of Civil Engineering, Liaoning Technical University, Fuxin 123000, China; zt_1987_zt@163.com (T.Z.); wanghongshan349@163.com (H.W.); 18940655383@163.com (X.Z.); 2 Key Lab of Structures Dynamic Behavior and Control of the Ministry of Education, Harbin Institute of Technology, Harbin 150000, China

**Keywords:** coal gangue concrete-filled circular steel tube columns, chloride salt corrosion, axial compressive bearing capacity, ultimate bearing capacity design method

## Abstract

The axial compressive behaviours of coal gangue concrete-filled steel tube (GCFST) columns after chloride salt corrosion were investigated numerically. Numerical modelling was conducted through the static analysis method by finite element (FE) analysis. The failure mechanism, residual strength, and axial load–displacement curves were validated against tests of the coal gangue aggregate concrete-filled steel tube (GCFST) columns at room and natural aggregate concrete-filled steel tube (NCFST) columns after salt corrosion circumstance. According to the analysis on the stress distribution of the steel tube, the stress value of the steel tube decreased as the corrosion rate increased at the same characteristic point. A parametric analysis was carried out to determine the effect of crucial variation on residual strength. It indicated that material strength, the steel ratio, and the corrosion rate made a profound impact on the residual strength from the FE. The residual strength of the columns exposed to chloride salt was in negative correlation with the corrosion rate. The impact on the residual strength of the column was little, obvious by the replacement rate of the coal gangue. A simplified design formula for predicting the ultimate strength of GCFST columns after chloride salt corrosion exposure was proposed.

## 1. Introduction

Incorporating coal gangue into concrete to replace natural aggregate provides an eco-friendly way to reuse coal mining and washing waste while reducing the production and consumption of natural aggregate [1,2]. However, the mechanical properties or durability of coal gangue aggregate is inferior to those of traditional concrete, because of the numerous surface microcracks, high porosity, and loose structure of coal gangue coarse aggregate. For example, replacing all coarse aggregate with coal gangue aggregate reduces the compressive strength of the concrete by 15–20% [3,4] (or 20–35% [5,6]), due to the porous structure of coal gangue, and the elastic modulus by 30–51% [7,8] while increasing the drying shrinkage by roughly 92% during a 360-day cure [9]. The rate of mass loss for natural aggregate concrete with 300 freeze–thaw cycles is only 5%, compared to over 5% for the rate of mass loss with 25 freeze–thaw cycles for concrete under a 60% replacement rate [10]. The above unfavorable results indicate that coal gangue concrete cannot be used for widespread application in building structures on a large scale.

A concrete-filled steel tube (CFST) is a new type of bearing structure made of a steel tube with concrete [11,12]. Under external load, the deformation of the core concrete is constrained by the steel tube, which can improve the deformability and compressive strength of the concrete. In the meantime, the core concrete can improve the stability of the steel tube and ensure the full play of the mechanical properties of steel. Therefore, CFSTs are widely used in building structures, underground engineering, bridge structures, oil drilling platforms, and transmission towers due to their high strength, outstanding seismic performance, good toughness, and convenient construction.

The behaviours of CFST columns with coal gangue aggregate (CGA) at room temperature have been extensively studied. Zhang et al. [13] studied the axial compression behaviours of the circular CFST stub prepared with CGA through experimental tests. A decreasing tendency was found in the elastic stiffness, compressive strength, and ductility of the columns by 16.17–8.24% (3.01–22.16%), 2.93–4.81% (8.25–10.18%), and 7.78–10.61% (11.86–16.34%), respectively, as the percentage of CGA increased from 0% to 50% (100%). Xu et al. [14] experimentally investigated the influence on the variation trend of the mechanical properties of square CFST columns by spontaneous combustion coal gangue aggregate (SCGA) especially under axial load. The experimental results showed that the peak compressive strength of the CFST columns was reduced by 5.96% and 12.10% at 50% and 100% of the SCGA replacement rate, respectively, compared to the CFST columns with traditional aggregate. Gao et al. [15] experimentally investigated and seriously analyzed the effects of coal gangue on the mechanical properties of different concrete, circular reinforced concrete columns, and CFST columns. The compressive strength properties of concrete with coal gangue reduced when the coal gangue replacement ratio increased, from the results. Fang et al. [16] presented a study on the seismic properties of ring-beam contact adopted for a connect beam using reinforced coal gangue concrete with a coal gangue concrete-filled steel tubular (GCFST) column.

Corrosion occurs frequently in the natural environment and also reduces the yield strength and durability of steel. Thus, it is essential that the residual behaviours of CFST columns after chloride salt corrosion be evaluated. Some studies have reported on the performance of GCFST columns after chloride salt corrosion. However, extensive studies have concerned the performance of natural aggregate concrete-filled steel tube (NCFST) members after salt corrosion, providing this research with a reference. According to the different corrosion regions, corrosion is divided into two types: uniform corrosion and local corrosion. 

For NCFST members exposed to salt corrosion with uniform corrosion, a lot of research has been conducted by Han et al. [17,18,19,20], Li et al. [21], Lyu et al. [22], Sultana et al. [23], Yuan et al. [24], Zeng et al. [25], Alraeeini et al. [26], Zhang et al. [27], Li et al. [28], Gao et al. [29], Reddy et al. [30], Wang et al. [31], and Zhang et al. [32]. 

In addition, there is some research concerning local corrosion behaviours, including studies on rectangular NCFST columns (Zhao et al. [33]), square NCFST columns (Guo et al. [34]), circular NCFST columns (Lin et al. [35], Zhao et al. [36], Luo et al. [37], Huang et al. [38], Karagah et al. [39], Wang et al. [40]), and L-shaped NCFST columns (Dinesh et al. [41]).

The above research results indicate that corrosion can cause the thickness of the outer steel tube in direct contact with the environment to become thinner, and the constraining ability of the steel tube on the core concrete is weakened [28,29,30,31,32]. Moreover, corrosion can also cause the deterioration of the yield strength and ductility of steel [42] and cause a decrease in ultimate strength, plastic deformation capacity, and combined elastic modulus. Thus, the study of the mechanical properties of the CFST specimen after chloride salt corrosion is one of the most popular subjects in the engineering field, and it is also an important issue for further studying the durability of CFST structures during service.

This paper thus focuses a numerical simulation on coal gangue concrete-filled circular steel tube (C-GCFST) stubs with uniform corrosion damage under axial compression. The main targets are as follows: (1) the development of an advanced finite element (FE) modelling method for simulating uniform corrosion damage in C-GCFST and the validation of the model against experimental data; (2) an analysis of the nonlinear behaviour of uniform corrosion damage in C-GCFST, including stress development and load–deformation relations; (3) a parametric sensitivity analysis to include identifying the key factors, e.g., material, replacement rate of coal gangue, and corrosion rate, on the residual strength of C-GCFST columns with corrosion; and (4) a proposal of a design formula for calculating the residual capacity of C-GCFST columns with corrosion exposure.

## 2. Finite Element Model and Experimental Verification

### 2.1. Methodology

The analysis results [43] show that the fiber model and the finite element analysis are the methods for the whole process of the analysis of the load–deformation relationship of CFST members. The fiber model is a simplified numerical analysis method, which assumes that the longitudinal stress at any point in the cross-section depends only on the longitudinal fiber strain at that point, but this analysis method is not conducive to further studying the working mechanism of CFST members from the perspectives of stress, strain distribution, and interaction between the steel tube and core concrete. The finite element analysis method can better solve this problem, and the interaction between the steel tube and the core concrete in the compressive process can be investigated in detail, which is conducive to revealing the mechanical essence of the CFST member more comprehensively. The long-term load effect is simulated by modifying the material’s constitutive relationship in the established model, and the “life-and-death element method” is used to simulate the corrosion effect of chloride ions on the steel tube. In simulating chloride corrosion, the corroded part of the unit is first cut into one or more layers of thin tube attached to the surface of the steel tube, and then the corroded part is meshed to make the corroded part become an independent unit. Once the set of corrosive elements is defined, a command is added to the analysis step where corrosion is taken into account, and the program that removes the sub-elements by gradually reducing the stiffness of the sub-elements to near zero is simulated in the calculation. The steel tube and concrete are simulated using a 3D solid with an eight-node reduced integral.

### 2.2. Material Constitutive

#### 2.2.1. Steel Tube

The *σ*-*ε* curve of the steel can be modelled using a quadratic flow plastic model, as shown in Equation (1) [42].

(1) $$\begin{array}{l} \sigma =\left\{\begin{array}{ll} E_{\mathrm{se}}\varepsilon & \;\;\varepsilon \leq \varepsilon_{e}\\ -A\varepsilon^{2}+B\varepsilon +C & \;\;\varepsilon_{e}<\varepsilon \leq \varepsilon_{e1}\\ f_{\mathrm{ye}} & \;\;\varepsilon_{e1}<\varepsilon \leq \varepsilon_{e2}\\ f_{\mathrm{ye}}\left[1+0.6\frac{\varepsilon -\varepsilon_{e2}}{\varepsilon_{e3}-\varepsilon_{e2}}\right] & \;\;\varepsilon_{e2}<\varepsilon \leq \varepsilon_{e3}\\ 1.6f_{\mathrm{ye}} & \;\;\varepsilon >\varepsilon_{e3} \end{array}\right. \end{array}$$

where *ε*_e_ = 0.8*f*_y_/*E*_s_, *ε*_e1_ = 1.5*ε*_e_, *ε*_e2_ = 10*ε*_e1_, *ε*_e3_ = 100*ε*_e1_, *A* = 0.2*f*_y_(*ε*_e1_ − *ε*_e_)^2^, *B* = 2*Aε*_e1_, and *C* = 0.8*f*_y_ + *Aε*^2^_e_ − *Bε*_e_. *f*_y_ and *E*_s_ are the yield strength and elastic modulus of the steel tube, respectively.

**Figure 1:**
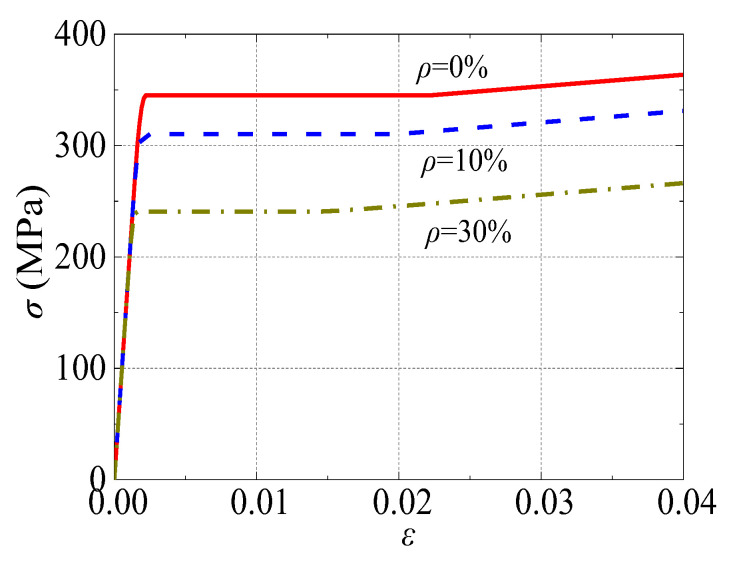
Stress-strain curve of Q345 steel after corrosion exposure.

The equations for calculating the mechanical properties of the steel tube as a function of the corrosion rate, according to Ref. [44], are shown in Equations (2) and (3). *f*_ye_ represents the tensile yield strength of the steel with various *ρ* (unit: MPa). *E*_se_ represents the elastic modulus of the steel tube with corrosion exposure. *ρ* is the corrosion rate (unit: %). The *σ-ε* relationship of Q345 steel under different *ρ* is presented in Figure 1.

(2) $$f_{\mathrm{ye}}/f_{y}=1-0.908\rho$$

(3) $$E_{se}/E_{s}=1-0.525\rho$$

#### 2.2.2. Concrete

Coal gangue concrete is a brittle material. The plastic damage relationship of concrete is used by ABAQUS 2022, which is applicable to coal gangue concrete materials with compressive and tensile anisotropy characteristics.

Due to incorporating coal gangue into the core concrete, which results in different properties from ordinary concrete, the compressive constitutive relationship of constrained concrete considering the replacement rate of coal gangue concrete aggregates proposed by Refs. [45,46] is adopted, as shown in Equations (4)–(9).

(4) $$\frac{\sigma }{\sigma_{0}}=\left\{\begin{array}{ll} 2\frac{\varepsilon }{\varepsilon_{0}}-{\left(\frac{\varepsilon }{\varepsilon_{0}}\right)}^{2} & \;\;\frac{\varepsilon }{\varepsilon_{0}}\leq 1\\ \frac{\left(\varepsilon /\varepsilon_{0}\right)}{\psi \beta {\left(\frac{\varepsilon }{\varepsilon_{0}}-1\right)}^{2}+\frac{\varepsilon }{\varepsilon_{0}}} & \;\;\frac{\varepsilon }{\varepsilon_{0}}>1 \end{array}\right.$$

where *ε*_0_ and *σ*_0_ are the peak strain and stress, respectively. *ε*_0_ is given by

(5) $$\varepsilon_{0}=(1300+12.5f_{c})\times 10^{-6}\lambda +800\times \xi^{0.2}\times 10^{-6}$$

where *f*_c_ is the compressive strength of coal gangue concrete (unit: MPa). *λ* is the influence of the coal gangue aggregate replacement fraction on strain, and *ξ* is the hoop coefficient, given by

(6) $$\xi =f_{\mathrm{ye}}A_{\mathrm{se}}/f_{c}A_{c}$$

where *f*_c_ is the compressive strength of coal gangue concrete (unit: MPa). *f*_ye_ is the yield strength after corrosion, and *A*_c_ and *A*_se_ are the cross-sectional area of the concrete and steel tube after corrosion, respectively, (unit: mm^2^), and

(7) λ=1+0.105r

where *r* is the coal gangue aggregate replacement fraction. From Equation (4), *ψ* is the influence of the coal gangue aggregate on descending section curvature.

(8) ψ=2.26−1.21r

Finally, *β* is the size of the area encompassed by the descending sections and the strain axis and is given by

(9) $$\beta =0.5\times {\left(2.36\times 10^{-5}\right)}^{\left(0.25+{\left(\xi -0.5\right)}^{7}\right)}\times f_{c}^{0.5}\geq 0.12$$

In the modelling of concrete, 53 is selected as the dilation angle, 0.1 is selected as the eccentricity, the ratio *f*_bo_/*f*_c_ is selected as 1.16, *K*_c_ is 0.66667, and 0.0005 is the viscosity parameter [47].

### 2.3. Finite Element Analysis

#### 2.3.1. Part and Meshing

In the finite element model’s establishment, a four-node linear reduction universal shell element (S4R) is selected for the steel tube. The reason is that S4R can determine whether to use thin shell theory or thick shell theory based on the thickness of it. The 9-integration point Simpson integration is set along the radial direction of the outer steel tube for the shell element. The 8-node reduced integral three-dimensional solid element (C3D8R) was selected for the core concrete.

To achieve reasonable calculation accuracy, the concrete and steel tube are longitudinally segmented, and the grid size of each component is controlled to be 0.01. Using structured partitioning techniques, the concrete is divided into hexahedral meshes.

#### 2.3.2. Interface Properties

Surface-to-surface contact is chosen in the interface properties between concrete and the steel tube. The hard contact is adopted in normal action, whilst the Coulomb friction model is selected in tangential action [48]. 

For the Coulomb friction model, shear stress can be transmitted between concrete and the steel tube. The relative sliding will be generated between the two materials as the value of the shear stress exceeds the critical value (*τ*_cnt_). At the same time, the shear stress at the inner surface of the steel tube will always be equal to *τ*_cnt_ during the sliding process. *τ*_cnt_ is positively correlated with the contacted pressure (*p*) between the steel tube and concrete surface, and the minimum value is not less than the average interfacial bonding force (*τ*_bond_). The calculation method for *τ*_cnt_ and *τ*_bond_ is shown in Equation (10), and *μ* is the cross-sectional friction coefficient, taken as 0.6 in this model.

(10) $$\tau_{\mathrm{cnt}}=\mu \cdot p\geq \tau_{\mathrm{bond}}$$

#### 2.3.3. Boundary Condition

**Figure 2:**
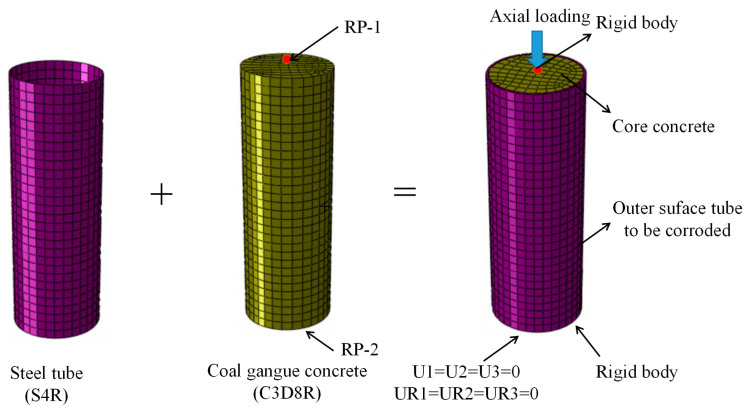
The established model of the C-GCFST stub column with uniform corrosion damage.

Set both ends of the specimen as a rigid body. The central point of the top and bottom rigid surface of the specimen is set to RP-1 and RP-2, respectively. The displacement of U1, U2, and U3 at the RP2 of the model is fixed; that is, the deformation of all of them is 0; U1, U2, and U3 represent the *X*-axis, *Y*-axis, and *Z*-axis displacement of the specimen cross-section, respectively. The bottom of the specimen is set as the loading end, and then limit the displacement in the U1 and U2 directions to 0; that is, the deformation of all of them except U3 is 0. A displacement loading regime is used to the model, and the numerical model is established in Figure 2.

### 2.4. Experimental Verification

Using ABAQUS 2022 finite element software, models of the C-GCFST stub column at room temperature [13,49] and the C-NCFST stub column after corrosion exposure [22,29] were established. Then, axial compressive bearing capacity was calculated. Finally, the simulated values of failure mode, load (*N*)-displacement (*∆*) curves, and the ultimate bearing capacity (*N*_u_) were analyzed, aiming at proving the accuracy of the model with experimental values. The equation for the corrosion of steel is based on Equation (11).

(11) $$\rho =\left(t_{0}-t_{1}\right)/t_{0}$$

where *ρ* represents the corrosion rate, (%). t_0_ represents the thickness of the tube before corrosion, (mm). t_1_ represents the thickness of the tube after corrosion (mm).

The corrosion experiment of the C-NCFST stub column was conducted by accelerated corrosion in the laboratory [22,29]. The mass fraction of the NaCl solution was 5%, and the PH value of the initial preparation solution was 3.0. The duration of the corrosion was set according to the different corrosion damage (5%, 10%, 20%, 30%). Afterward, the 200 t [22,29] and 500 t [13,49] hydraulic compression machine was used for the compressive tests. The total eight strain gauges were placed symmetrically at the middle height of each specimen as well as linear variable differential transformers (LVDTs) recording axial displacement. A rate of 2.0 kN/s was set before the load reached 0.7 *N*_u_; after that, the loading interval was 1/15 *N*_u_, and a rate of 1.0 kN/s was designed during the compression. In the post-peak stage, the loading rate was adopted for displacement control by 0.5 mm/min until the compressive stub column failed [13]. The test was set for displacement control by a loading rate of 1 mm/min until the axial displacement reached 20 mm [49].

#### 2.4.1. Failure Pattern

**Figure 3:**
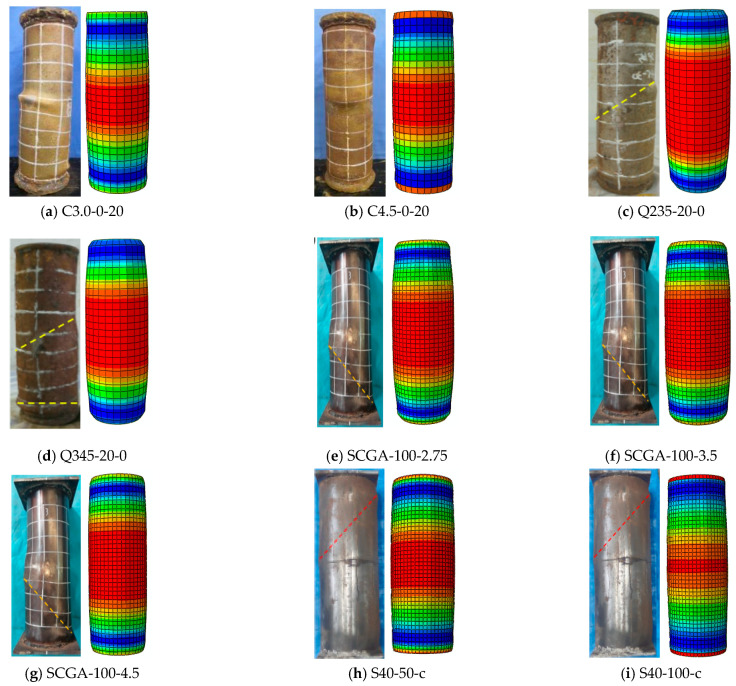
The numerical and experimental failure pattern of the typical C-CFST stub columns. (**a**) C3.0-0-20; (**b**) C4.5-0-20; (**c**) Q235-20-0; (**d**) Q345-20-0; (**e**) SCGA-100-2.75; (**f**) SCGA-100-3.5; (**g**) SCGA-100-4.5; (**h**) S40-50-c; (**i**) S40-100-c.

Due to the insignificant effect of corrosion and the coal gangue replacement rate on the failure mode of stub columns, a comparison of the failure modes of the typical specimens is provided in Figure 3. The dotted lines represent shear-slip lines of failure specimens.

From the FE results, it is said that the failure mode of the specimen is mainly bulging outward along the height direction of the specimens. At the end plate and middle part of the specimen height, the bulging of the column is more obvious. The bulging value of the column at the same cross-section is the same. In the Ref. experiment, the middle of the stubs and the end plate both produced significant bulging, and the bulging values of the column at the same cross-section were different, indicating that the overall failure mode was shear failure. The reason for this phenomenon is that the premise of FE analysis is to assume that the steel and concrete are isotropic, and it cannot be guaranteed that the specimen is in a fully axial compression state in actual compression tests. The effectiveness of the FE model established in this method was confirmed by comparing the failure modes. 

#### 2.4.2. Load (*N*)-Displacement (*∆*) Curve

**Figure 4:**
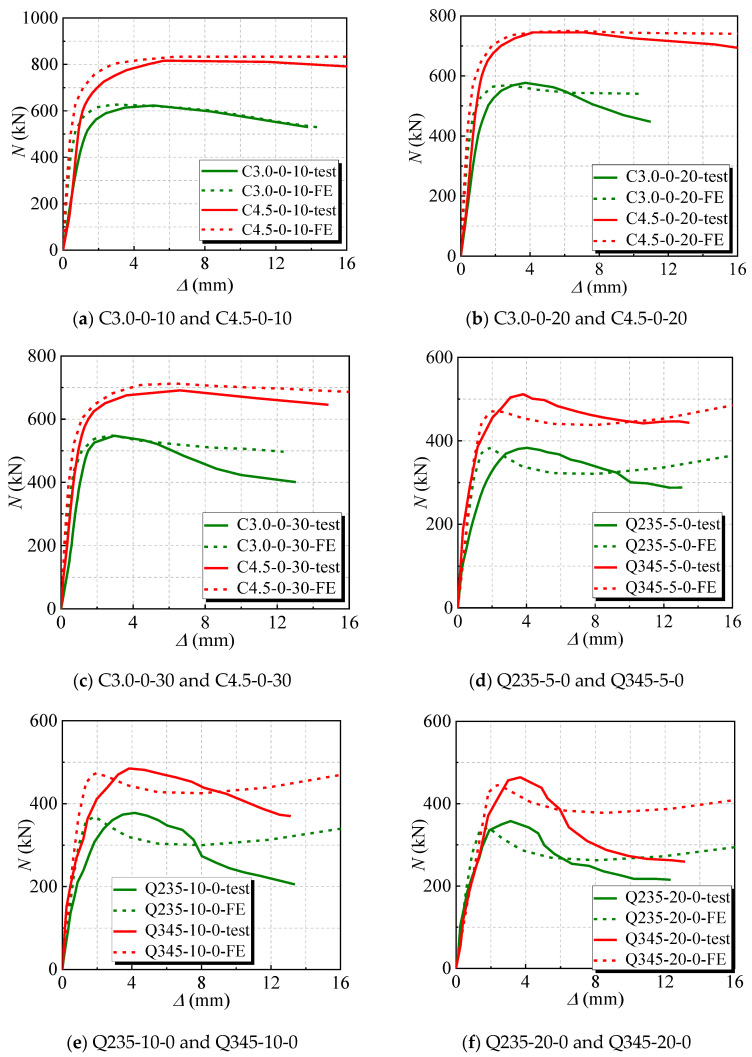
The compared numerical and experimental N-∆ curves of the C-CFST stub after chloride salt corrosion. (**a**) C3.0-0-10 and C4.5-0-10; (**b**) C3.0-0-20 and C4.5-0-20; (**c**) C3.0-0-30 and C4.5-0-30; (**d**) Q235-5-0 and Q345-5-0; (**e**) Q235-10-0 and Q345-10-0; (**f**) Q235-20-0 and Q345-20-0.

**Figure 5:**
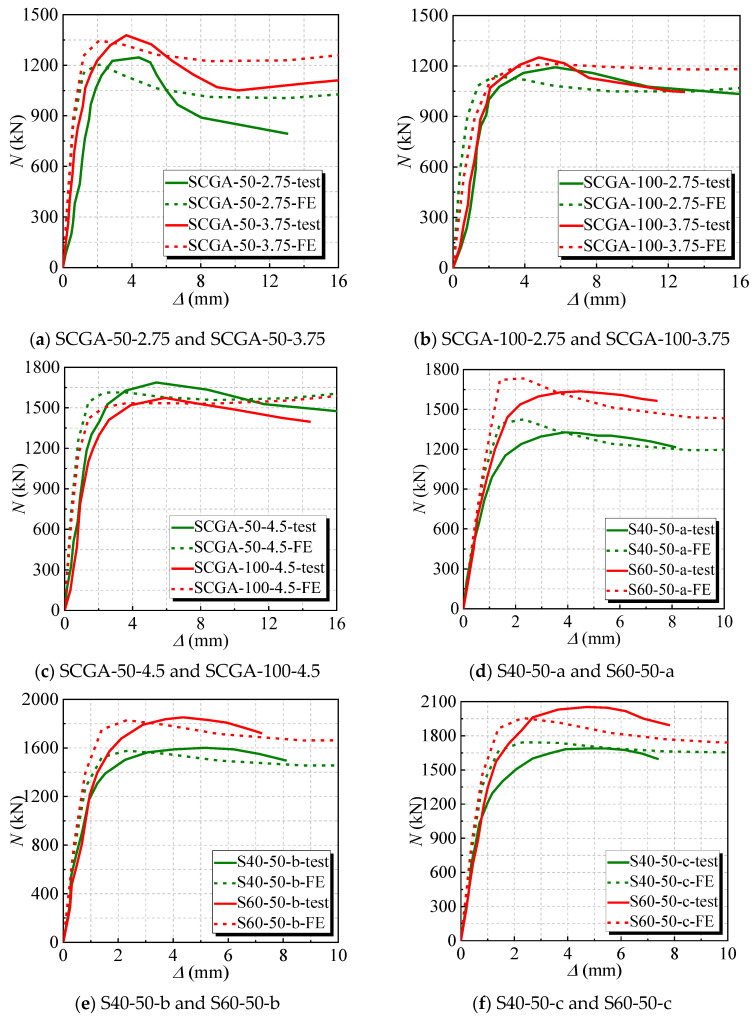

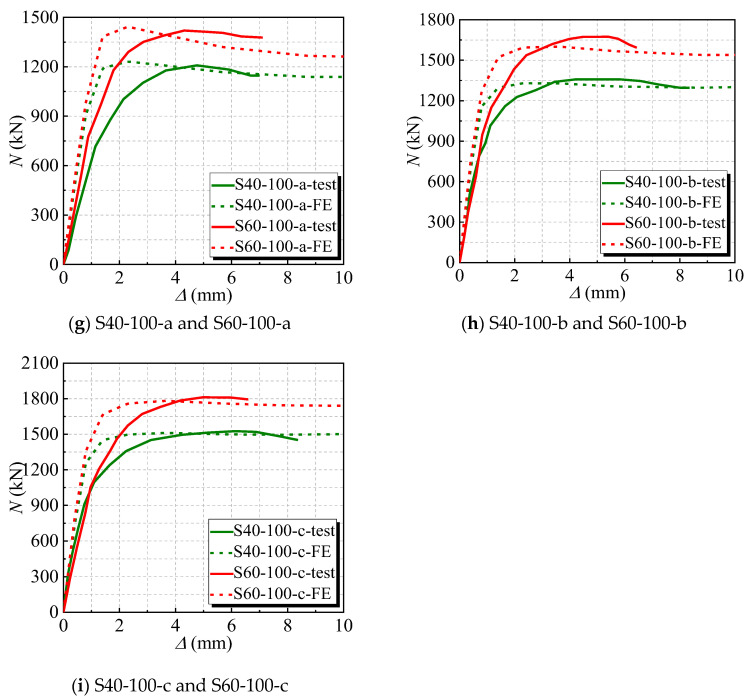
The compared numerical and experimental *N–∆* curves of the C-GCFST stub at room temperature. (**a**) SCGA-50-2.75 and SCGA-50-3.75; (**b**) SCGA-100-2.75 and SCGA-100-3.75; (**c**) SCGA-50-4.5 and SCGA-100-4.5; (**d**) S40-50-a and S60-50-a; (**e**) S40-50-b and S60-50-b; (**f**) S40-50-c and S60-50-c; (**g**) S40-100-a and S60-100-a; (**h**) S40-100-b and S60-100-b; (**i**) S40-100-c and S60-100-c.

The results between the experimental and numerical *N*-*∆* curves are compared for the stub columns under axial load and the measured curve, as shown in Figure 4 and Figure 5. It is said that the initial stiffness of the numerical *N*-*∆* curves of several stubs is greater than that of the experimental curves. Possibly, when FE simulation is carried out, the boundary-constrained cross-section of the stubs is set in a perfectly fixed condition, which are more idealized and have relatively powerful constraint effects, resulting in a higher value for initial stiffness. Overall, the trends of the two curves are roughly the same, and they match well, indicating that the finite element model is reasonable.

#### 2.4.3. Ultimate Bearing Capacity

The numerical values (*N*_f_) and experimental values (*N*_e_) of the peak load of the stub are presented in Table 1. The ratio of numerical values to experimental values is between 0.929 and 1.074, with a mean of 0.990, an SD of 0.033, and a CV of 0.033. It indicates that the finite element method is relatively accurate. *N*_f_ represents the numerical values, and *N*_e_ represents the experimental values in the References.

## 3. Numerical Investigations

The stress mechanism of the C-GCFST columns with uniform corrosion damage under axial compression throughout the stressing process is analyzed further by the finite element software (ABAQUS 2022) adopted, including the *N*-*∆* curves of the columns, stress distribution in the steel tube, and the interaction mechanism between the outer steel tube and core concrete. The key parameters of a characteristic specimen are selected as follows: the section diameter (*D*) is 400 mm, concrete strength (*f*_cu_) is 30 MPa, yield strength of the steel (*f*_y_) is 345 MPa, replacement rate (*r*) is 50%, corrosion rate (*ρ*) is 0, 10%, and 30%, and steel ratio *θ* is 3%.

### 3.1. Load (N)-Deformation (∆) Curve

**Figure 6:**
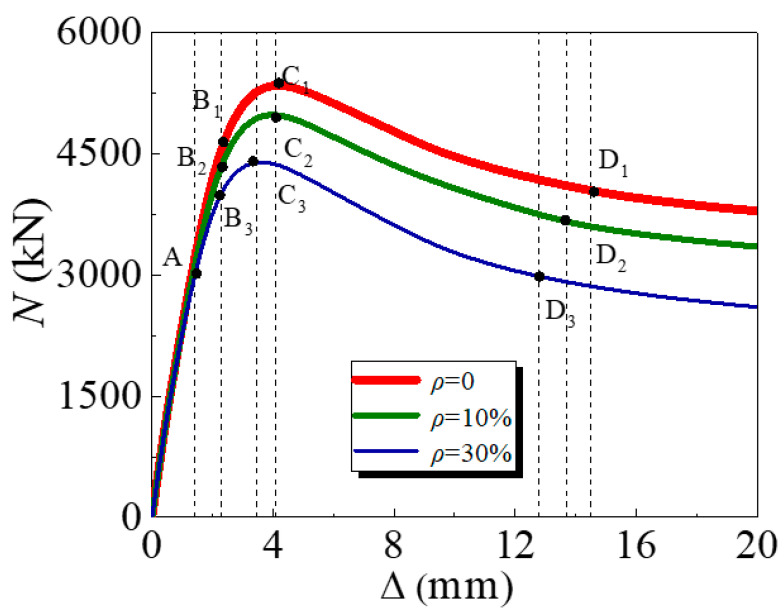
Load–displacement curves.

The *N*-*∆* curves of the specimens after corrosion exposure are presented in Figure 6, and it is said that *N*_u_ significantly decreases with corrosion. According to the variation tendency of the *N*-*∆* curve, four characteristic points are defined, namely point A when the concrete is constrained by the steel tube, B at the yield point when the steel tube yields, point C when the specimen reaches the ultimate strength, and point D when *N*_u_ reduces and tends to stabilize. The load-displacement curve is divided into five segments by four characteristic points, with the OA section being the elastic stage, the AB section is in the elastic–plastic stage, the BC section is in the plastic strengthening stage, and the CD section is the descending segment.

In the OA section, the GCFSTs are in separate compression states. For the outer steel tube, the tensile stress reaches the proportional limit at the middle region of the column height as the load reaches point A. At this time, the compressive loads of the three specimens are 58.5%, 61.1%, and 67.1% of their ultimate loads, respectively, and the longitudinal displacement is 1.4 mm. 

In the AB section, when the load exceeds point A, the compressive specimen begins to turn into the plastic deformation stage, and the *N*-*∆* curve of the specimen exhibits nonlinearity. The rate of load increasing slows down, the longitudinal displacement increases faster, and the axial force on the concrete increases continually. At this point, core cracks of the concrete appear and expand continually, and Poisson’s ratio of coal gangue concrete exceeds gradually compared to that of the steel tube. The contact pressure between the two materials also increases continuously. The specimen is at the critical point of elasticity and plasticity as the load reaches point B, and the loads of the three specimens are 86.9%, 88.9%, and 93.2% of their ultimate loads, respectively. The longitudinal displacement is 2.3 mm.

In the BC section, as the load exceeds point B, the increase in load tends to slow down, and the increase in axial displacement continues to accelerate. When the load approaches point C, the *N*-*∆* curve tends to flatten, and the ultimate strength of the specimen is reached, with a longitudinal displacement of 3.7 mm.

When the load exceeds point C, the externally tensile steel tube has already yielded, and the inner coal gangue concrete has reached its ultimate compressive strain. Subsequently, as the longitudinal displacement of the stubs increases, the concrete stress decreases, and the overall stiffness of the specimen decreases, meaning that it can no longer bear axial loads, and the load begins to decrease. 

After the load reaches point D, as deformation increases, the speed of load reduction slows down, which also indicates that the compressive stress of the columns tends to stabilize. The loads of the three specimens are 72.9%, 71.9%, and 70.0% of their ultimate loads, respectively. The longitudinal displacement is 12.8 mm, 13.7 mm, and 14.5 mm, respectively.

### 3.2. The Stress Distribution of the Steel Tube

**Figure 7:**
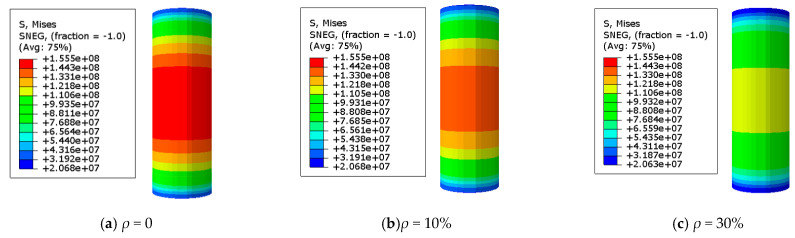
Mises stress distribution of the steel tube at point A. (**a**) *ρ* = 0; (**b**) *ρ* = 10%; (**c**) *ρ* = 30% (unit: Pa).

**Figure 8:**
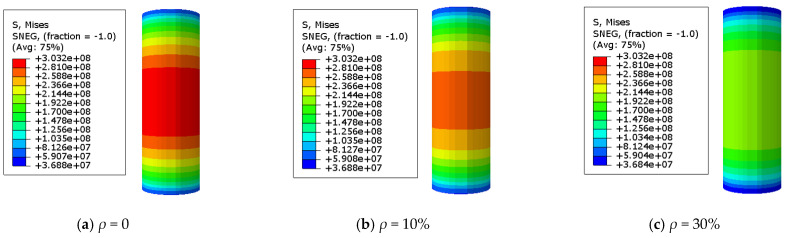
Mises stress distribution of the steel tube at point B. (**a**) *ρ* = 0; (**b**) *ρ* = 10%; (**c**) *ρ* = 30% (unit: Pa).

**Figure 9:**
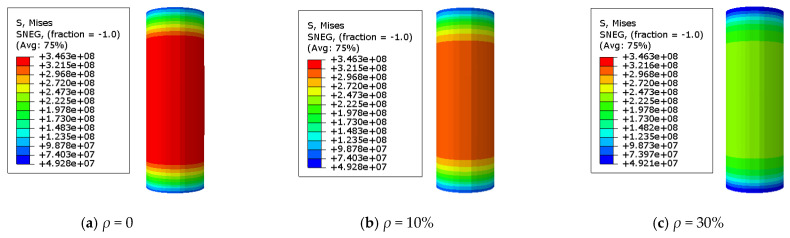
Mises stress distribution of the steel tube at point C. (**a**) *ρ* = 0; (**b**) *ρ* = 10%; (**c**) *ρ* = 30% (unit: Pa).

**Figure 10:**
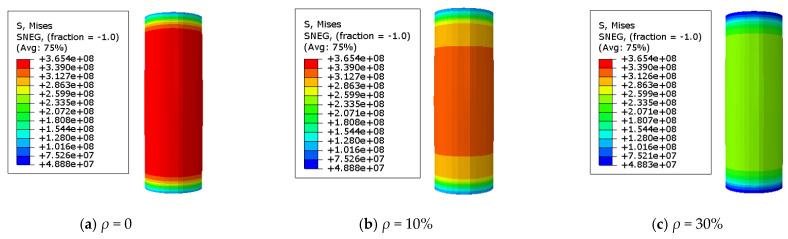
Mises stress distribution of the steel tube at point D. (**a**) *ρ* = 0; (**b**) *ρ* = 10%; (**c**) *ρ* = 30% (unit: Pa).

The adequate analysis of Mises stress in the specimen is beneficial for quickly identifying the hazardous area of stub columns. A Mises stress cloud map of the C-GCFST stub columns after corrosion exposure is found at points A, B, C, and D from Figure 7, Figure 8, Figure 9, and Figure 10, respectively. An elastic state can be seen in the compressive specimen before point A, with a relatively uniform stress distribution along the longitudinal direction of the steel tube and lower stress at both ends. During the loading exerted from point A to point B, the Mises tensile stress values of the steel tube increase slowly. At point B, the tensile stress at the longitudinal middle region of the compressive steel tube reaches *f*_y_. At point C, the stress on the compressive steel tube still maintains a uniform distribution. Due to the defined reinforced section of the steel after corrosion exposure, compared to point B, when the outer steel tube stress reaches the yield stress, the maximum stress of the steel tube at point C improves, and the yield region of the steel tube also increases. After point D, the cross-section of the column undergoes significant expansion and deformation. Under the same characteristic points, the stress value of the steel tube decreases with the corrosion rate increasing.

## 4. Parameter Analysis

**Figure 11:**
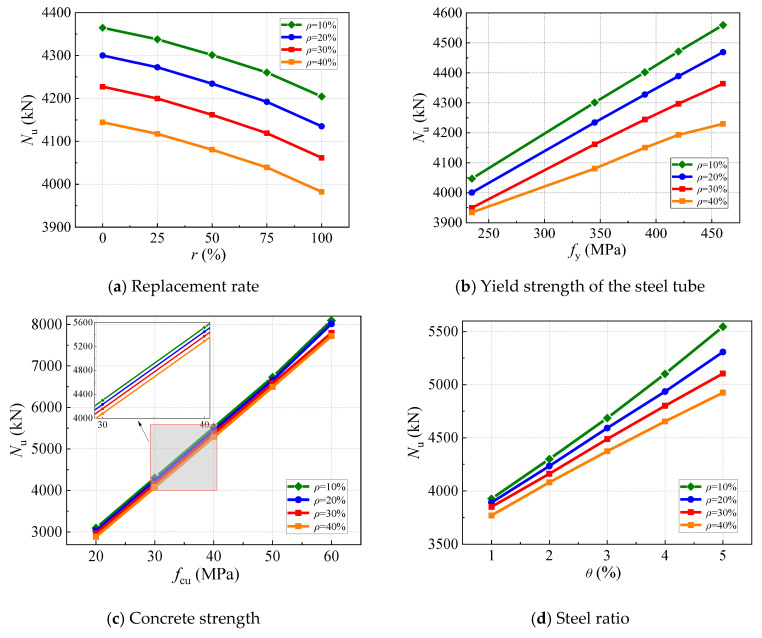
The influence of variations on Nu: (**a**) replacement rate; (**b**) the yield strength of the steel tube; (**c**) concrete strength; and (**d**) steel ratio.

The considered parameters included concrete strength (*f*_cu_), the yield strength of the steel tube (*f*_y_), the replacement rate (*r*), corrosion rate (*ρ*), and steel ratio (*θ*). The ranges of these variations are presented in Table 2. The ultimate bearing capacity of the C-GCFST stub after corrosion exposure (*N*_u_) is analyzed in detail by different variations, as shown in Figure 11.

The influence of *r* on *N*_u_ is presented in Figure 11a. Other parameters remain unchanged, but the ultimate bearing capacity of the stub decreases as the *r* of the coal gangue aggregate increases. Taking the corrosion rate of 10% as a sample for analysis, the *r* of the coal gangue aggregate increases from 0 to 25%, 50%, 75%, and 100% with *N*_u_ decreasing by 0.62%, 1.46%, 2.39%, and 3.67%, respectively. And the rate of decline becomes faster and faster. The reason may be that the mechanical and surface characteristics of the coal gangue aggregate are poor compared to the natural aggregate. The compressive properties of the coal gangue concrete gradually decrease as the proportion of the coal gangue aggregate increases, resulting in a reduction in *N*_u_.

In addition, while keeping the *r* of the coal gangue aggregate unchanged, the negative correlation can be predictable between *N*_u_ and *ρ*. For specimens with an *r* of 0, 25%, 50%, 75%, or 100%, *N*_u_ decreases by 5.04%, 5.08%, 5.13%, 5.19%, and 5.28% with the *ρ* increasing from 10% to 40%, respectively. This is because the specimen is immersed in a solution rich in Cl^−^, which causes the external steel tube to electrolyze Fe^2+^ and Fe^3+^, causing the corrosion and thinning of the outer wall for the corroded steel tube, thereby weakening the constrained effective coefficient of the outer steel tube on the restrained concrete and reducing *N*_u_.

The influence of *f*_y_ on *N*_u_ is presented in Figure 11b. For specimens with a corrosion rate of 10%, *N*_u_ increases by 6.28%, 8.77%, 10.49%, and 12.67% with the increase in the *f*_y_ of the steel tube from 235 MPa to 345 MPa, 390 MPa, 420 MPa, and 460 MPa, respectively. The reason is that the higher the yield strength of the steel tube, the stronger the constrained effect of the outer steel tube on the core concrete, resulting in an increase in the value of *N*_u_. When the *f*_y_ of the steel tube (235 MPa, 345 MPa, 390 MPa, 420 MPa, 460 MPa) is the fixed value, *N*_u_ decreases by 2.78%, 5.13%, and 5.72% with the *ρ* increasing from 10% to 40%, 6.22%, and 7.23%, respectively. This indicates that promoting the *f*_y_ of the outer steel tube will not reduce the loss of corrosion on the compressive bearing capacity of the specimen.

The effect of *f*_cu_ on *N*_u_ is shown in Figure 11c. When the concrete strength grade (20 MPa, 30 MPa, 40 MPa, 50 MPa, 60 MPa) is the fixed value, *ρ* increases from 10% to 40%, and *N*_u_ reduces by 6.66%, 5.16%, 4.16%, 3.47%, and 4.62%, respectively. This indicates that the corrosion rate has a slight effect on the compressive bearing capacity of the columns. The reason is that concrete affords a high contribution rate to the compressive bearing capacity of the columns, and the core concrete is not affected by corrosion.

The influential effect of the steel ratio (*θ*) on *N*_u_ is presented in Figure 11d. For specimens with a corrosion rate of 10%, *θ* is enhanced from 2% to 3%, 4%, and 5%, and *N*_u_ is promoted by 9.51%, 19.34%, 29.95%, and 41.24%, respectively. This is because as *θ* increases, the wall thickness of the used steel tube is thicker at the same diameter. Under the same *ρ*, the constrained concrete more restrained by a thicker steel tube is found, which can improve *N*_u_. When *θ* (2%, 3%, 4%, 5%) is the fixed value, *N*_u_ decreases by 4.01%, 5.10%, 6.66%, 8.79%, and 11.19% with *ρ* increasing from 10% to 40%, respectively. It may be because *ρ* has a more significant destructive effect on the steel tube, and increasing the steel ratio of the specimen is not enough to repair the deficiency of the specimen.

## 5. Simplified Design Method

We propose a simplified method for predicting the ultimate bearing capacity of C-CFST columns undergoing corrosion that is based on Chinese Standard GB50936-2014 [50], Technical Code for Concrete-Filled Steel Tubular Structures and Zhang et al. [44], as given by the following:

(12) $$N_{0}=A_{\mathrm{sc}}f_{\mathrm{sc}}$$

where *N*_0_ is the unmodified design strength of the C-CFST columns (N), *A*_sc_ is the total cross-section (mm^2^), and *f*_sc_ is the compressive strength for the C-CFST columns (MPa). *f*_sc_ is given by

(13) $$f_{\mathrm{sc}}=\left(1.212+B\xi +C\xi^{2}\right)f_{c}$$

where

(14) $$B=\frac{0.176f_{\mathrm{ye}}}{213}+0.974$$

(15) $$C=\frac{-0.104f_{c}}{14.4}+0.031$$

and *ξ* the hoop coefficient of C-CFST is

(16) $$\xi =\theta_{\mathrm{se}}\frac{f_{\mathrm{ye}}}{f_{c}}$$

with *f*_ye_ as the yield strength of the steel tube after corrosion, *f*_c_ the compressive strength of a concrete cylinder, and *θ*_se_ the steel ratio after corrosion exposure. *f*_ye_ is given by

(17) $$f_{\mathrm{ye}}=\left(1-0.908\rho \right)f_{y}$$

where *ρ* is the corrosion rate, and *f*_y_ is the yield strength of the uncorroded steel tube. *f*_c_ is given by

(18) $$f_{c}=\left(0.76+0.2\mathrm{lg}\left(\frac{f_{\mathrm{cu}}}{19.6}\right)\right)f_{\mathrm{cu}}$$

where *f*_cu_ is the compressive strength of a concrete cube. *θ*_se_ is given by

(19) $$\theta_{\mathrm{se}}=\frac{A_{\mathrm{se}}}{A_{c}}$$

where *A*_se_ and *A*_c_ are the cross-section of the steel tube and concrete after exposure, respectively.

**Figure 12:**
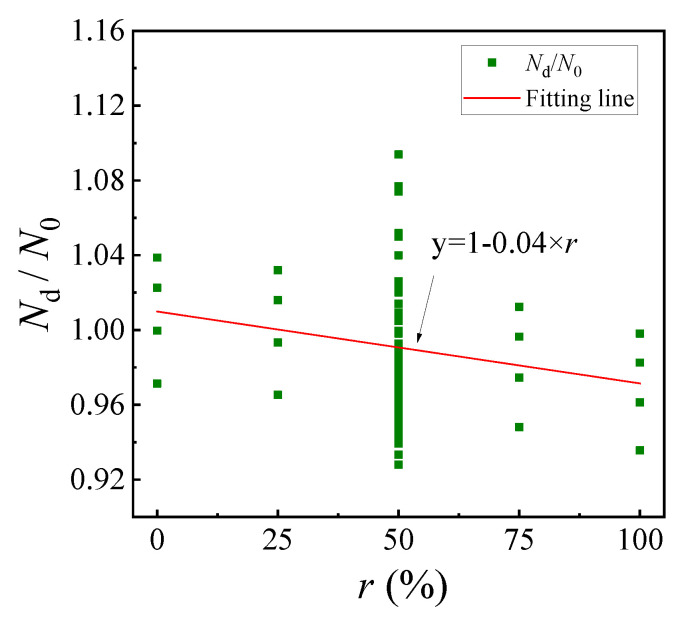
The curve of *N*_d_/*N*_0_ and *r*.

Since the design strength of the C-GCFST stub columns with corrosion exposure will be affected by the coal gangue coarse aggregate, the design method for predicting the design strength of C-GCFST columns with corrosion is modified according to the relationship between *N*_d_/*N*_0_ and *r* in Figure 12.

(20) $$N_{d}=N_{0}\left(1-0.04\times r\right)$$

where *r* presents the replacement rate of coal gangue.

**Figure 13:**
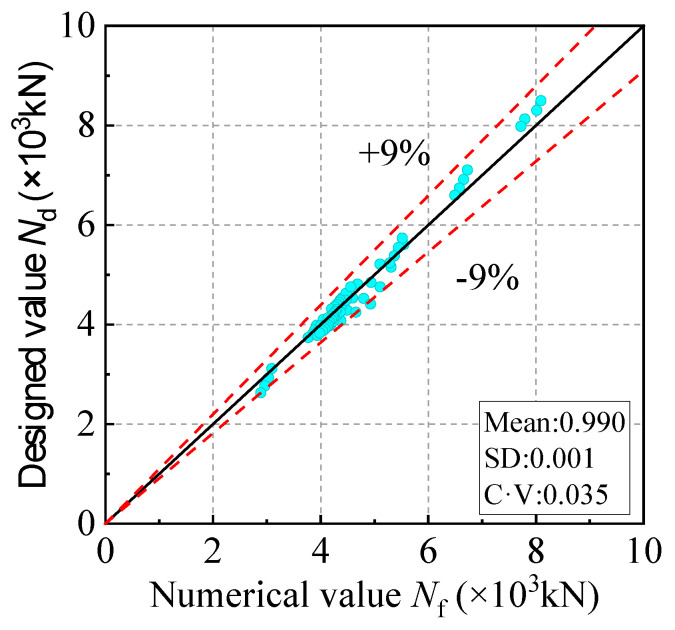
Results of *Nd* and *Nf*.

The numerical values (*N*_f_) and designed values (*N*_d_) of the design strength of the C-GCFST columns with corrosion exposure are shown in Figure 13. The error between *N*_f_ and *N*_d_ is within 9%, with a mean of 0.990, an SD of 0.001, and a CV of 0.035, which indicates that the predictive effect of this formula is reasonable.

## 6. Conclusions

The mechanical response of the C-GCFST stub columns was investigated by establishing a numerical model, especially in uniform corrosion damage. According to the simulated results, the presented conclusions are as follows:

(1) Compared to the FE and Ref. experimental results, it is said that the failure mode of them was shear failure with bulging outward along the height direction of the specimens. The ratio of numerical to experimental values is between 0.929 and 1.074, with a mean of 0.990 and an SD of 0.033. This indicates that the finite element method is relatively accurate.
(2) When the load exceeds the steel yield strength, as the corrosion rate increases, the specimen will enter various characteristic regions. At the same characteristic points, the stress value of the steel tube decreases with increasing corrosion rate due to the lower bearing capacity of the specimen.
(3) The concrete strength, steel yield strength, and steel ratio are positively correlated with the compressive bearing capacity of the specimen. The increase in the steel yield strength and steel ratio will not reduce the loss from corrosion on the compressive bearing capacity of the stub.
(4) The corrosion rate and replacement rate are negatively correlated with the *N*_u_ of the specimen. When the *r* of the coal gangue aggregate increases from 0 to 100%, *N*_u_ decreases by 3.67%, 3.84%, 3.92%, and 3.91% due to worse mechanical properties of coal gangue, respectively, within the parameter range of this study.
(5) A design method was proposed for predicting the design strength of C-GCFST stub columns with corrosion. The error between the numerical values and designed values is within 9%, which indicates that the predictive effect of this formula is reasonable.

## Figures and Tables

**Table 1:** Comparison between numerical and experimental values.

No.	*D* (mm) × *L* (mm) × *t* (mm)	*ρ* (%)	*E*_s_ (GPa)	*f*_ye_ (MPa)	*f*_ue_ (MPa)	*μ*_s_	*N*_f_ (kN)	*N*_e_ (kN)	*N*_f_/*N*_e_	Ref.
C3-0-10	90 × 300 × 3.0	10	152	359	431	0.282	623	623	1.011	[22]
C3-0-20		20	134	288	339	0.296	570	577	0.987	
C3-0-30		30	122	229	325	0.283	549	548	1.002	
C4.5-0-10	90 × 300 × 4.5	10	145	339	403	0.266	833	816	1.021	
C4.5-0-20		20	140	305	336	0.279	750	745	1.007	
C4.5-0-30		30	128	258	318	0.309	713	691	1.031	
Q235-5-0-1/2	90 × 270 × 1.78	5	208	242	474	-	384	383	1.003	[29]
Q235-10-0-1/2		10	208	242	474	-	369	381	0.969	
Q235-20-0-1/2		20	208	242	474	-	341	354	0.965	
Q345-5-0-1/2	90 × 270×1.90	5	210	359	531	-	490	511	0.960	
Q345-10-0-1/2		10	210	359	531	-	474	485	0.978	
Q345-20-0-1/2		20	210	359	531	-	447	464	0.963	
SCGA-50-2.75	140 × 420 × 2.75	-	198	278	346	0.258	1207	1246	0.969	[13]
SCGA-100-2.75		-	198	278	346	0.258	1139	1179	0.966	
SCGA-50-3.75	140 × 420 × 3.75	-	205	285	364	0.252	1347	1384	0.973	
SCGA-100-3.75		-	205	285	364	0.252	1213	1306	0.929	
SCGA-50-4.50	140 × 420 × 4.50	-	201	338	420	0.262	1614	1657	0.974	
SCGA-100-4.50		-	201	338	420	0.262	1535	1549	0.991	
S40-50-a	156 × 450 × 3.0	-	201	282	459	0.28	1425	1327	1.074	[49]
S40-100-a		-	201	282	459	0.28	1232	1201	1.026	
S60-50-a		-	201	282	459	0.28	1734	1630	1.064	
S60-100-a		-	201	282	459	0.28	1443	1420	1.016	
S40-50-b	158 × 450 × 4.0	-	206	295	465	0.28	1577	1659	0.951	
S40-100-b		-	206	295	465	0.28	1330	1368	0.972	
S60-50-b		-	206	295	465	0.28	1828	1865	0.980	
S60-100-b		-	206	295	465	0.28	1600	1672	0.957	
S40-50-c	159 × 450 × 4.5	-	204	317	477	0.29	1744	1718	1.015	
S40-100-c		-	204	317	477	0.29	1511	1540	0.981	
S60-50-c		-	204	317	477	0.29	1957	2039	0.960	
S60-100-c		-	204	317	477	0.29	1782	1812	0.984	
Mean value									0.990	
SD									0.033	
CV									0.033	

Note: *D* represents the section diameter of the specimens. *L* represents the length of the specimens. *t* represents the steel tube wall thickness. *E*_s_ represents the elastic modulus. *f*_ye_ and *f*_ue_ represent the yield strength and ultimate strength. *μ*_s_ represents Poisson’s ratio. *N*_f_ represents the numerical value of the ultimate strength. *N*_e_ represents experimental ultimate strength. SD represents the standard deviation. CV represents the coefficient of variation. Mean value represents the mean value of all *N*_f_/*N*_e_.

**Table 2:** Parameters of C-GCFST stub for parametric analysis.

Parameter	Ranges	Default
*f*_cu_ (MPa)	20, 30, 40, 50, 60	30
*f*_y_ (MPa)	235, 345, 390, 420, 460	345
*θ* (%)	1, 2, 3, 4, 5	2
*r* (%)	0, 25, 50, 75, 100	50
*ρ* (%)	10, 20, 30, 40	—

## Data Availability

The raw data supporting the conclusions of this article will be made available by the authors on request.

## Nomenclature

*A*_c_	cross-section area of concrete	*N*_d_	modified ultimate bearing capacity with replacement rate
*A*_s_	cross-section area of steel	*N*_u_	ultimate bearing capacity
*A*_s__e_	cross-section area of steel after exposure	*N*_f_	peak load of the numerical model
*A*_sc_	total column cross-section	*N*_e_	peak load of the experiment
*D*	section diameter of the specimen	*μ*	cross-section friction coefficient
*L*	length of the specimen	*r*	coal gangue aggregate replacement fraction
*E*_c_	elastic modulus of concrete	*μ*_s_	Poisson’s ratio
*E*_s_	elastic modulus of steel tube	*t*_0_	thickness of steel tube before corrosion
*E*_se_	elastic modulus of steel tube with corrosion exposure	*t*_1_	thickness of steel tube after corrosion
*f*_s__c_	compressive strength of the CFST column	*θ*	steel ratio
*ξ*	hoop coefficient of the CFST	RP-2	bottom point of the model
*f*_c_	compressive strength of the coal gangue concrete cylinder	*θ*_se_	steel ratio after corrosion exposure
*f*_cu_	compressive strength of concrete cubes	*p*	contacted pressure in finite element model
*f*_b0_	compressive strength of concrete under biaxial loading	*β*	size of the area encompassed by the descending sections and the strain axis
*f*_y_	yield strength of steel tube	*Ψ*	influence of the coal gangue on descending curvature
*f*_ye_	yield strength of steel tube with corrosion exposure	*λ*	influence of the coal gangue substitute fraction on strain
*f*_ue_	ultimate strength of steel tube with corrosion rate	*τ*_bond_	bonding force in finite element model
*ρ*	corrosion rate	*τ*_cnt_	critical value in finite element model
*N*	axial load	*σ*	stress
*∆*	axial displacement	*σ*_0_	peak stress
*N*_0_	unmodified ultimate bearing capacity	*ε*	strain
*K*_c_	compressive meridian	*ε*_0_	peak strain
U1, U2, U3	*X*-, *Y*-, *Z*-axis displacement	*ε*_e_	elastic strain
UR1, UR2, UR3	*X*-, *Y*-, *Z*-axis angle of rotation	*ε*_e1_, *ε*_e2_, *ε*_e3_	process strain of the compression
RP-1	top point of the model		
